# Carcinoid Tumor: Advances in Treatment Options

**DOI:** 10.7759/cureus.6641

**Published:** 2020-01-13

**Authors:** Urwat T Vusqa, Stuti Patel, Mamoon Ur Rashid, Deepika Sarvepalli, Abu H Khan

**Affiliations:** 1 Internal Medicine, Army Medical College, Rawalpindi, PAK; 2 Internal Medicine, Gujarat Medical Education and Research Society Medical College, Vadodara, IND; 3 Internal Medicine, AdventHealth, Orlando, USA; 4 Internal Medicine, Guntur Medical College, Guntur, IND; 5 Gastroenterology, AdventHealth, Orlando, USA

**Keywords:** carcinoid tumor, neuroendocrine tumors, small bowel cancer, lower gastrointestinal bleed

## Abstract

Small bowel neoplasms are rare, accounting for only 3%-6% of all gastrointestinal neoplasms. Carcinoid tumors represent a large portion of these (20%-30%), making them the second most common small bowel malignancy after adenocarcinoma. Gastrointestinal carcinoids constitute 70% of all neuroendocrine tumors, and out of those, 42% originate in the small bowel. They are predominantly seen in older patients around the age of 65 years. From 1973 to 2004, there has been more than a fourfold increase in the incidence of carcinoid tumors. This can be probably due to increased diagnostic accuracy rather than an actual increase in the number of new cases. The workup of a suspicious case of gastrointestinal bleeding consists of esophagogastroduodenoscopy and/or colonoscopy, and other imaging tests including video capsule endoscopy and balloon-assisted endoscopy. Management of the tumors is dependent on the size and location of the lesion. Treatment options include surgery, endoscopic removal of tumors, and various immunotherapy and chemotherapeutic agents.

## Introduction

Small bowel neoplasms are uncommon, accounting for only 3%-6% of all gastrointestinal (GI) neoplasms. Carcinoid tumors represent the second most common (20%-30%) small bowel malignancy after adenocarcinoma. GI carcinoids constitute 70% of all neuroendocrine tumors (NETs), and out of those, 42% originate in the small bowel [1,2]. They are predominantly seen in older patients around the age of 65 years [3]. The incidence of NETs is rare, constituting 0.5% of all newly diagnosed malignancies [4].

From 1973 to 2004, there has been more than a fourfold increase in the incidence of carcinoid tumors [5]. This can be probably due to increased diagnostic accuracy rather than an actual increase in the number of new cases [2]. We present a case of a carcinoid tumor that presented with GI bleed and anemia. The patient had normal findings on esophagogastroduodenoscopy and colonoscopy and was provisionally diagnosed with malignancy on computerized tomography (CT) abdomen/pelvis with contrast.

## Case presentation

A 52-year-old otherwise healthy man presented to his primary care physician with complaints of bleeding per rectum for the past six days. He described the stools as dark in color and pasty in consistency. He also complained of colicky right lower quadrant abdominal pain. Physical examination was unremarkable except for mild generalized abdominal pain. He was found to have a hemoglobin of 9 mg/dl, and was referred to the emergency room for evaluation. Further lab studies revealed iron deficiency anemia, and fecal occult blood was positive. Further, the patient underwent a CT abdomen/pelvis with contrast, which showed a 3.7 x 3.7 x 3.2 cm mesenteric mass in the right mid-abdomen with associated desmoplastic reaction and thickening of the surrounding distal ileum, highly suspicious of carcinoid tumor. The patient's serotonin and chromogranin levels were also reported to be elevated.

**Figure 1:**
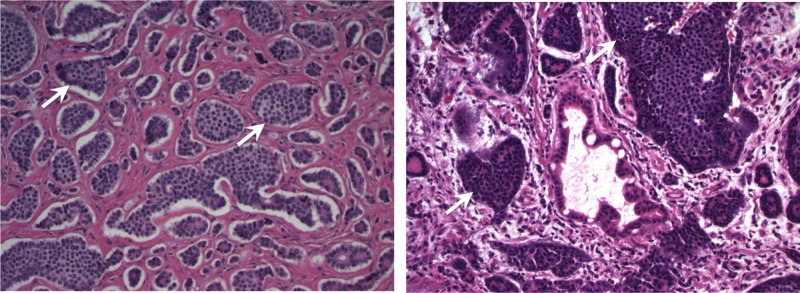
Multiple foci of grade 1 (G1) well-differentiated NET extending into serosa and involving mesenteric margins NET: neuroendocrine tumor

The patient was then started on venofer for his anemia. To locate the source of bleeding, the patient underwent esophagogastroduodenoscopy and colonoscopy. However, both tests failed to demonstrate a source of an active or recent GI bleed. The options of attempted biopsy with interventional radiology versus surgical exploration with resection of affected area and mass were discussed with the patient. Considering the patient's symptoms and iron deficiency anemia, surgery was performed with the patient's informed consent. The patient completed the course of venofer and underwent explorative laparotomy with segmental ileal resection and primary reanastomosis. Gross findings were significant for a large abnormal mass of mesentery of distal ileum with dilated and thickened overlying small bowel. There was no evidence of peritoneal implants or liver masses. Resected mass and tissue were sent for pathology. The pathology report confirmed the presence of multiple foci of well-differentiated grade 1 (G1) NET extending to the serosa and involving the mesenteric margins (Figure 1). Three out of ten lymph nodes were positive for metastatic NET with the largest lymph node measuring 1-2 cm in diameter. The tumor stage was consistent with pT4 and pN1. The early postoperative period was unremarkable, and the patient was recommended to follow up with surgery and oncology.

## Discussion

Small bowel, which constitutes 75% of the total length and 95% of the total surface area of the GI tract, is not a usual source for GI neoplasms due to multiple reasons. The contents of the small bowel are dilute and less irritating to the mucosa, and the transit time is rapid, which means less carcinogenic exposure. Besides, the bacterial count is low, implying that organisms may not be able to convert proto-oncogenes to oncogenes. Also, the small bowel has an abundance of immunoglobulin A containing lymphoid tissue [6]. Of all the small bowel tumors, carcinoid is of particular significance since it can present with obscure gastrointestinal bleeding (OGIB). OGIB, which represents about 5% of lower GI bleeding, is defined as active GI bleeding where the cause cannot be explained after a normal esophagogastroduodenoscopy and colonoscopy result [7]. The source of such bleed is usually the small bowel, and the common causes vary with age, as shown in Table 1 [7].

**Table 1:** Most common causes of obscure gastrointestinal bleeding CD: Crohn's disease; GIST: gastrointestinal stromal tumor; AVM: arteriovenous malformation

Age of the patient	Common cause of obscure gastrointestinal bleeding
< 20 years	Meckel diverticulum, CD
20-60 years	Small bowel tumors (GIST, carcinoid), CD, AVM
> 60 years	AVM, small bowel tumor (GIST, carcinoid)

The common causes of lower GI bleeding are shown in Table 2 [8].

**Table 2:** Common causes of lower GI bleeding GI: gastrointestinal

Causes of lower GI bleed
1. Upper GI causes
Ulcer
Neoplasm
Vasculitis
2. Small bowel causes
Inflammatory bowel disease
Neoplasm
Arteriovenous malformation
Meckel diverticulum
Mesenteric ischemia
Radiation induced
Infections
Aortoenteric fistula
3. Colorectal causes
Diverticular disease (most common)
Neoplasm
Inflammatory bowel disease
Angiodysplasia
Ischemic colitis
Aortoenteric fistulas
Post-polypectomy
Infections
Radiation induced

NETs of small bowel usually remain asymptomatic for a long time or can present with ill-defined symptoms [2]. The manifestation of the disease becomes more prominent when the tumor spreads to the liver (40%), or the mass results in acute complications like bowel obstruction, perforation, bleeding, or ischemia [3]. Local complications are due to the hormones produced by the primary tumor, which further leads to widespread mesenteric and peritoneal fibrosis [9]. Metastatic disease to the liver often presents with systemic symptoms such as diarrhea, wheezing, and flushing due to the production of vasoactive peptides like serotonin, vasoactive intestinal peptide, and histamine [10,11]. A retrospective analysis was conducted in Massachusetts on adult patients with histologically proven small bowel NETs. In that study, the majority of the patients presented with OGIB and anemia (87.5%) [12], and the leading cause of mortality was liver failure (80%) followed by bowel obstruction in 16% of the cases [9].

**Figure 2:**
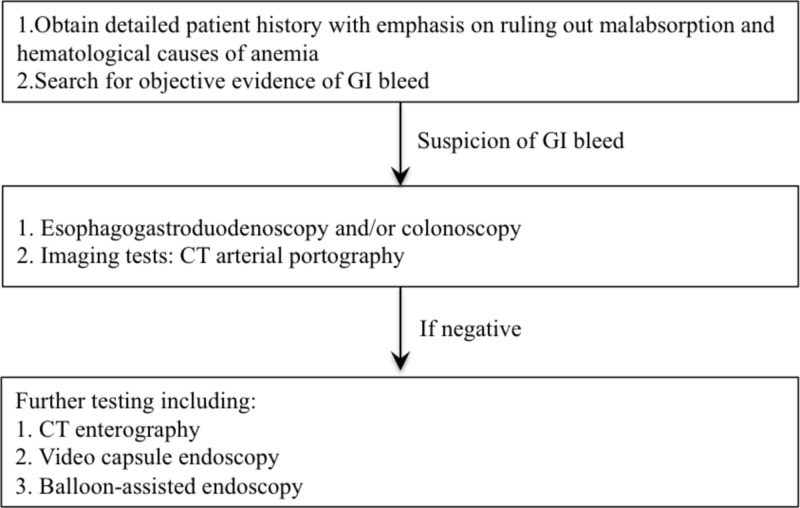
ACG guidelines for obscure GI bleeding ACG: The American College of Gastroenterology; GI: gastrointestinal; CT: computerized tomography

The initial workup of NETs consists of a CT and magnetic resonance imaging of the abdomen/pelvis [12,13]. However, despite having good sensitivity and specificity, their role in diagnosing NETs of the small bowel is often limited [13]. In the past decade, video capsule endoscopy (VCE) has emerged as an important test in diagnosing small bowel NETs [12,14]. In a retrospective analysis by Noujaim et al., the findings on VCE, including tumor location, morphology, and number, correlated with the findings obtained during surgery [12]. Furthermore, a meta-analysis by Triester et al. done on 88 patients with OGIB revealed that VCE was able to detect the source of bleeding in 67% of the patients [14]. These results are in contrast to the 8% of cases detected by small bowel radiographic studies. A study by Hakim et al. proposed that CT enterography can help identify NETs that were missed on VCE [15]. However, there is still some discrepancy between what is detected on imaging and what is observed intraoperatively [3]. NETs are usually diagnosed late, with the majority presenting with metastases to liver and lymph nodes (85.5%) [3]. Interestingly, the primary tumor often remains small. The American College of Gastroenterology guidelines in approaching a case of obscure GI bleeding is shown in Figure 2 [16,17].

Management of the tumors is dependent on the size and location of the lesion. Duodenal NETs that are < 1 cm can be removed through an endoscope as they are often isolated masses with no metastasis. In contrast, jejunoileal tumors often require surgery and present with early metastasis to lymph nodes and liver [2]. Ki-67 index, a histopathological marker, helps in tumor grading. A higher index signifies an aggressive tumor growth. According to the World Health Organization (WHO) classification of 2017, grade 1/grade2 (G1/G2) tumors have a Ki-67 index of 0%-20%, and grade 3 (G3) has an index of >20% (Table 3) [3,18]. For G1 tumors, regardless of liver involvement, surgical resection of the primary tumor with radical lymph node dissection has shown survival benefit [3,9]. In contrast, for G2 tumors, data are less supportive of surgery benefit, and it is usually not recommended unless for symptomatic palliative relief [3]. For liver metastasis, management consists of surgical resection, even if the entire metastatic tumor cannot be eradicated. This is due to the fact that NETs produce vasoactive amines that precipitate liver failure (the leading cause of mortality), and tumor resection helps in preventing or at least slowing down the progression of liver failure [9]. In a cohort study conducted in Germany, the investigators observed that patients who had an R0 or R1 resection of their hepatic metastasis fared better with a five-year survival rate of 88.5% compared to 69.1% five-year survival rate in those who underwent R2 resection or no liver resection at all [3].

**Table 3:** WHO 2017 grading of NET WHO: World Health Organization; NET: neuroendocrine tumor; G: grade; HPF: high-power field

WHO 2017 grading of NETs		
Tumor grade	Mitoses/10 HPF	Ki-67 index
Well differentiated		
G1	<2	<3
G2	2 to 20	3 to 20
G3	>20	>20
Poorly differentiated		
G3	>20	>20

Currently, immunotherapy is emerging to be a thriving option in the management of patients with GI and pancreatic NETs [19]. In the past, somatostatin analogs and interferon-alpha have shown to improve the quality of life and survival in these patients [20]. Various immunoproteins like cytotoxic T-lymphocyte-associated protein 4 (CTLA-4) and programmed cell death-1 (PD-1)/programmed death-ligand 1 (PD-L1) are being targeted to reveal their benefits in patients with GI NETs [19]. Biotherapy is still emerging, and it is expected to unveil better benefits not only for NETs but also for many other cancers. Several clinical trials are underway, focusing on the potential benefits of biotherapy alone and in combination with chemotherapy as well (Table 4) [20].

**Table 4:** List of various biotherapy clinical trials PD-1 pathway: programmed death-1 pathway; CTLA-4: cytotoxic T-lymphocyte-associated protein 4; CTL: cytotoxic T-lymphocyte; mTOR: mammalian target of rapamycin; PARP: poly(ADPribose)polymerase; EGFR: epidermal growth factor receptor

Study	Trial phase	Drug	Mechanism of action	Conditions
Ibrutinib in advanced carcinoid and pancreatic neuroendocrine tumors	Phase II	Ibrutinib	Inhibits Bruton's tyrosine kinase and hence B-cell receptor signaling	Carcinoid tumors, pancreatic neuroendocrine tumors
Fosbretabulin in subjects with pancreatic or GI neuroendocrine tumors with elevated biomarkers	Phase II	Fosbretabulin tromethamine	Destabilizes microtubules and targets tumor vasculature thus inhibiting angiogenesis	Neuroendocrine tumors
Nivolumab with ipilimumab in subjects with neuroendocrine tumors	Phase II	Nivolumab and ipilimumab	Inhibits immune responses through PD-1 pathway. ipilimumab binds to CTLA-4 and blocks the normal inhibitory signal thus allowing CTLs to destroy cancer cells	Neuroendocrine tumors, carcinoid tumors
The MetNET-2 trial (MetNET-2)	Early phase I	Lanreotide and metformin	Lanreotide: somatostatin analog; metformin anti-diabetic agent, recently emerged as anti-tumor agent; acts through modification of systemic metabolism or through cell pathway effects	Neuroendocrine tumors
Nintedanib in patients with locally advanced or metastatic neuroendocrine tumors	Phase II	Nintedanib	Inhibits growth factors through inhibition of tyrosine kinase	Carcinoid tumor, metastatic carcinoid tumor, neuroendocrine neoplasms
Everolimus and octreotide in patients with advanced carcinoid tumor (RADIANT-2)	Phase III	Octreotide vs octreotide+everolimus	Octreotide: somatostatin analog, everolimus: inhibits mTOR	Carcinoid tumor, malignant carcinoid syndrome
Carfilzomib in patients with advanced neuroendocrine cancers	Phase II	Carfilzomib	Selectively inhibits proteosomes	Neuroendocrine cancers
Combination chemotherapy plus interferon-alpha followed by filgrastim in patients with GI tumors	Phase II	Biological agent: filgrastim Recombinant interferon-alpha drugs: fluorouracil and hydroxyurea	Filgrastim: colony-stimulating factor; helps body make white blood cells. Recombinant interferon-alpha: therapeutic peptide, at molecular level to exert anti-viral and anti-tumor effects. Flourouracil: anti-metabolite, anti-neoplastic agent. Hydroxyurea: anti-metabolite, slows cancer growth	Extrahepatic bile duct cancer, gastric cancer, gastrointestinal carcinoid tumor, liver cancer, pancreatic cancer, small intestine cancer
Telotristat etiprate-expanded treatment for patients with carcinoid syndrome symptoms (TELEPATH)	Phase III	Telotristat etiprate tablets	Inhibits tryptophan hydroxylase	Carcinoid syndrome
Veliparib, capecitabine, and temozolomide in patients with advanced, metastatic, and recurrent neuroendocrine tumor	Phase I	Capecitabine, temozolomide, veliparib	Capecitabine: anti-metabolite, anti-cancer drug. Temozolomide: alkylating anti-cancer drug. Veliparib: inhibits protein PARP; acts at molecular level by blocking PARP and thus allow cancer cells to respond better to other chemotherapy agents	Functional pancreatic neuroendocrine tumor, metastatic carcinoid tumor, somatostatin-producing neuroendocrine tumors
Erlotinib hydrochloride and cetuximab in patients with advanced gastrointestinal cancer, head and neck cancer, non-small cell lung cancer, or colorectal cancer	Phase I	Cetuximab, erlotinib hydrochloride	Cetuximab: EGFR inhibitor; erlotinib: EGFR (tyrosine kinase) inhibitor	Metastatic gastrointestinal carcinoid tumors, recurrent colon cancer, recurrent gastrointestinal carcinoid tumors

## Conclusions

Small bowel carcinoids are rare tumors that present with vague symptoms like GI bleed and anemia. A thorough investigation is required in any case of suspicious GI bleed, especially in older patients over 65 years. Management consists of surgery and various chemotherapy and immunotherapy drugs. Biotherapy is still emerging, and it is expected to unveil better benefits not only for NETs but also for many other cancers.
